# International Trends in Antidepressant Consumption: a 10-year Comparative Analysis (2010–2020)

**DOI:** 10.1007/s11126-025-10122-0

**Published:** 2025-03-03

**Authors:** Alberto Peano, Francesco Calabrese, Konstantinos Pechlivanidis, Riccardo Mimmo, Gianfranco Politano, Manuela Martella, Maria Michela Gianino

**Affiliations:** 1 Department of Public Health Sciences and Paediatrics, University of Turin, Via Santena 5/Bis, 10126 Turin, Italy; 2 Department of Control and Computer Engineering, Polytechnic of Turin, 10138 Turin, Italy; 3 Medical direction, Santa Croce e Carle Hospital, Cuneo, Italy; 4 Medical direction, ASL Alessandria, Alessandria, Italy

**Keywords:** Antidepressant consumption, OECD trends, Mental health disorders, Antidepressant drugs

## Abstract

The present study aims to analyze country-specific antidepressant consumption rates across 30 OECD countries, comparing them with the OECD average from 2010 to 2020. This analysis examines annual variations in consumption and identifies potential trends over time. Using a retrospective observational design, the study employs pooled secondary data from 30 OECD countries. A time trend analysis was conducted to assess overall and country-specific linear and quadratic trends, modeling the variation in Defined Daily Dose (DDD) per 1,000 inhabitants from 2010 to 2020. Findings reveal a significant increase in antidepressant consumption across most countries. The mean DDD value rose from 52.42 in 2010 to 69.5 in 2020. Three distinct trend patterns emerged: linear, concave, and convex. While 15 countries exhibited a significant linear increase, five showed a plateauing trend, and others experienced exponential growth with a convex trajectory. Denmark was the only country to show a decline in consumption, whereas Norway displayed no significant change over time. These results underscore a notable rise in antidepressant use across most OECD countries, alongside variations in consumption trajectories. By assessing and comparing national consumption rates, this study highlights the importance of international collaboration in addressing mental health challenges and optimizing healthcare accessibility and outcomes.

**Supplementary Information:**

The online version contains supplementary material available at 10.1007/s11126-025-10122-0.

## Introduction

Antidepressants are among the most widely prescribed medications worldwide, with their usage increasing significantly over the years. Several drug classes fall under the category of antidepressants, with Selective Serotonin Reuptake Inhibitors (SSRIs), Tricyclic Antidepressants (TCAs), Monoamine Oxidase Inhibitors (MAOIs), and Heterocyclic Agents (HCAs) being the main groups [1, 2].

The progressive rise in antidepressant consumption can be attributed to the introduction of newer, safer, and more tolerable molecules, such as SSRIs. The expanding use of SSRIs has been facilitated by a better understanding of the serotonin neurotransmitter’s role and mechanism of action [3]. Due to their improved tolerability, SSRIs have been approved for additional indications, such as neuropathic pain and alcoholism, making them the most prescribed class of antidepressants [2, 4–6].

However, the increasing use of antidepressants is driven by multiple factors within a complex landscape. Key contributors include advancements in clinical practice (e.g., improved recognition of psychiatric symptoms and conditions), the superior safety profile of newer drugs, greater patient acceptability and adherence, and the frequent recommendation for long-term treatment [7]. Despite these advantages, data on the long-term safety of antidepressants remain incomplete. The increasing consumption, coupled with potential underestimation of the balance between benefits and risks, poses a public health concern with long-term implications [8, 9]. Furthermore, antidepressant prescriptions for children and adolescents have steadily risen despite ongoing debates and controversies regarding their use in these age groups [10, 11].

Currently, many antidepressants are available as generics, and in numerous countries, the sales of generic equivalents surpass those of branded formulations. Switching from brand-name to generic antidepressants is considered a cost-effective strategy. However, despite the increasing consumption of generics, overall spending on antidepressants has continued to rise over time [12, 13].

Previous studies on antidepressant use have primarily focused on factors influencing their consumption, trends within individual countries [14–16], or specific geographical regions over different time periods [3, 17]. However, there is a lack of comparative research examining antidepressant consumption trends across multiple countries.

In this context, the present study aims to compare antidepressant consumption trends across multiple countries and assess relative changes during 2020–2021. Specifically, we analyze antidepressant consumption rates in 30 member countries of the Organization for Economic Cooperation and Development (OECD), using the OECD average as a benchmark. Additionally, we evaluate annual variations in antidepressant consumption to determine whether these changes remained consistent. Such analyses are crucial for public health monitoring, as they provide insights into the prevalence of mental health conditions and their societal impact. Understanding antidepressant consumption trends can enhance resource allocation, ensure an adequate supply of mental health services, and identify cost implications. Finally, these trends can serve as indicators of the effectiveness of interventions and policies, while also aiding in the evaluation of prescription appropriateness and adherence to clinical guidelines.

## Methods

This retrospective observational study analyzed pooled secondary data from 30 OECD countries to assess time trends in antidepressant consumption from 2010 to 2020. Antidepressants were classified according to the Anatomical Therapeutic Chemical (ATC) system under the code N06A. Consumption was measured as Defined Daily Dose (DDD) per 1,000 inhabitants, defined as “the assumed average maintenance dose per day for its main indication in adults” (https://data-explorer.oecd.org/). Annual country-specific consumption data were extracted from the OECD Data Explorer Database. Whenever possible, the dataset reflects total consumption, including hospital use. The sources and methodologies for data collection are specified for each country and are accessible via the OECD Data Explorer under the "Pharmaceutical Consumption" section in "Health."

### Statistical Analysis

A linear model was used to analyze country-specific linear and quadratic trends and assess variations in DDD per 1,000 inhabitants from 2010 to 2020. The model included centered years and squared-centered years as covariates. The coefficient (b) represents the linear trend, with a positive or negative value indicating an increasing or decreasing trend, respectively. The b-squared coefficient captures the curvature of a non-linear relationship. A positive b-squared coefficient suggests a convex trend (where growth or decline accelerates over time), while a negative b-squared coefficient suggests a concave trend (where growth or decline slows over time). To further analyze trends, the annual mean DDD value was calculated, and both linear and quadratic trends were assessed. Additionally, a multilevel linear regression model with random intercept and random slope was applied to estimate fixed and random effects for the annual DDD of antidepressants per 1,000 inhabitants. This model was chosen to account for the clustered nature of the data, allowing intercepts and slopes to vary across countries.

MS Excel © was used for data management. Regression for trend estimation has been modelled with the linear model function of the stats package in R 4.3.1 and StataSE 18. Data are publicly accessible on the OECD health data website (https://data-explorer.oecd.org/). Therefore, approval from the ethical committee is not required.

## Results

### DDD per 1,000 Inhabitants

**Fig. 1:**
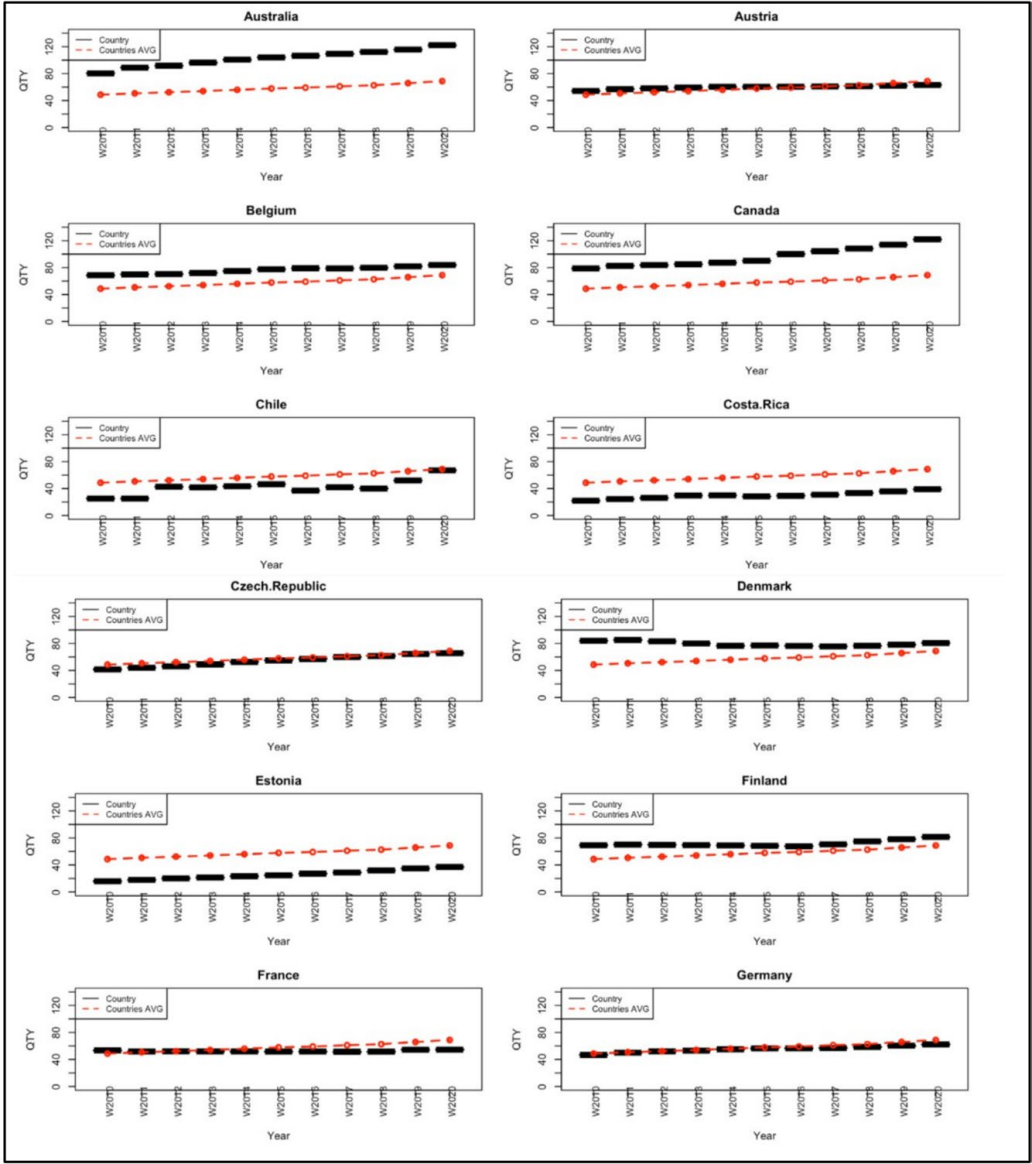

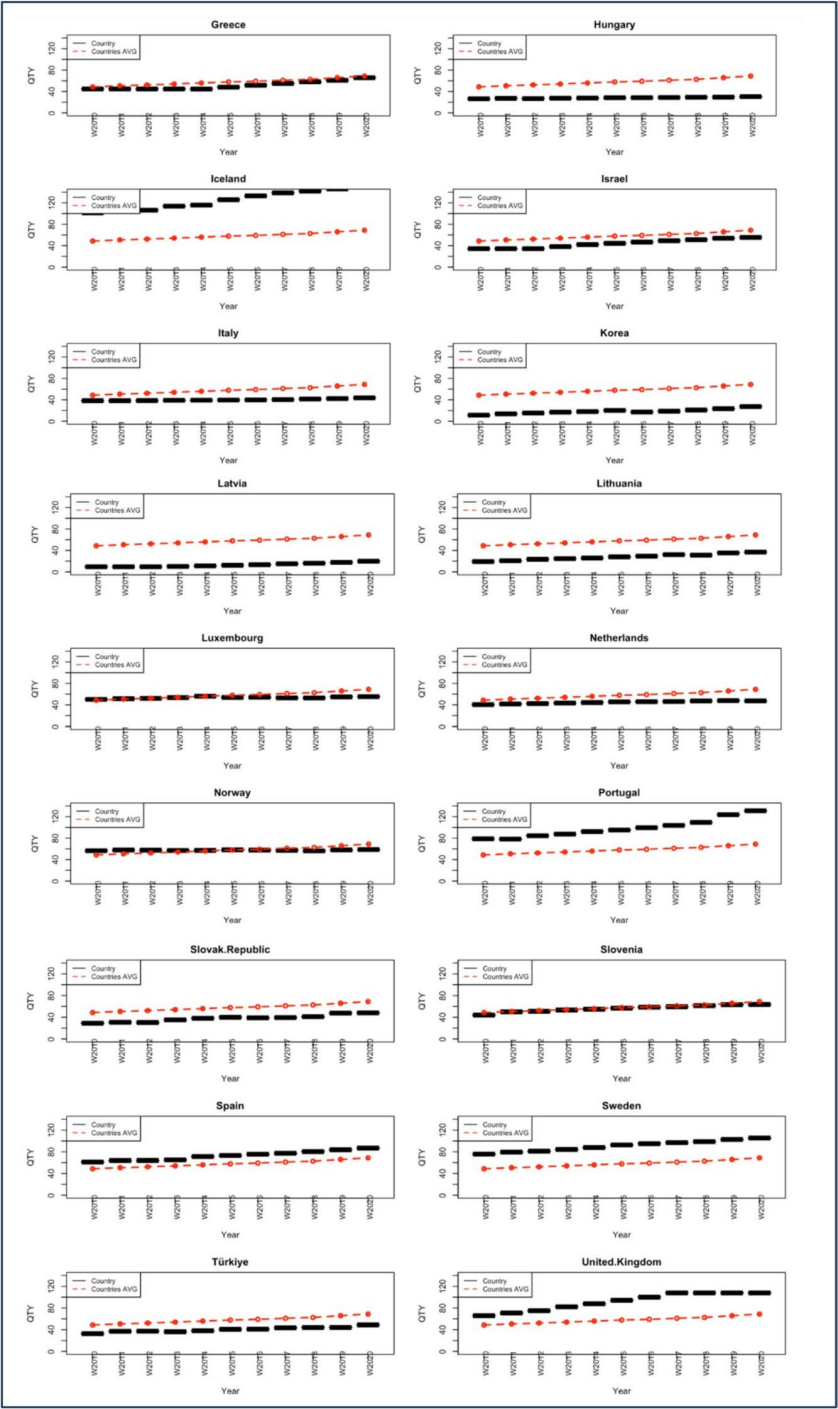
Country-specific antidepressant consumption /1000 inhabitants (black dashed line) during 2010–2020 compared to the average OECD value (red dash-dotted line). Abbreviations: *AVG* Average; *QTY* Quantity; *OECD* Organization for Economic Cooperation and Development. Quantity is measured in DDD per 1000 inhabitants; DDD means Defined Daily Doses, namely the assumed average maintenance dose per day, according to pharmacological indication for adults. The black dashed line refers to country-specific consumption in DDD between 2010 and 2020, while the red dash-dotted line refers to the average values computed among 30 OECD countries, also measured in DDD during the same timeframe

Country-specific DDD per 1,000 inhabitants per year was compared with the yearly OECD average (see Fig. 1). Based on consumption levels over the 11-year period, the evaluated countries were categorized into three groups:

1. Countries with consistently higher consumption than the OECD average (Australia, Austria, Belgium, Canada, Denmark, Finland, Iceland, Portugal, Spain, Sweden and the United Kingdom).
2. Countries with consistently lower consumption than the OECD average (Chile, Costa Rica, Czech Republic, Estonia, Germany, Greece, Hungary, Israel, Italy, Latvia, Lithuania, Luxembourg, Korea, Netherlands, Slovak Republic, Slovenia and Turkey).
3. Countries where consumption was above the OECD average in 2010 but fell below it by 2020 (France and Norway).

### Annual Trend in DDD per 1,000 Inhabitants

**Fig. 2:**
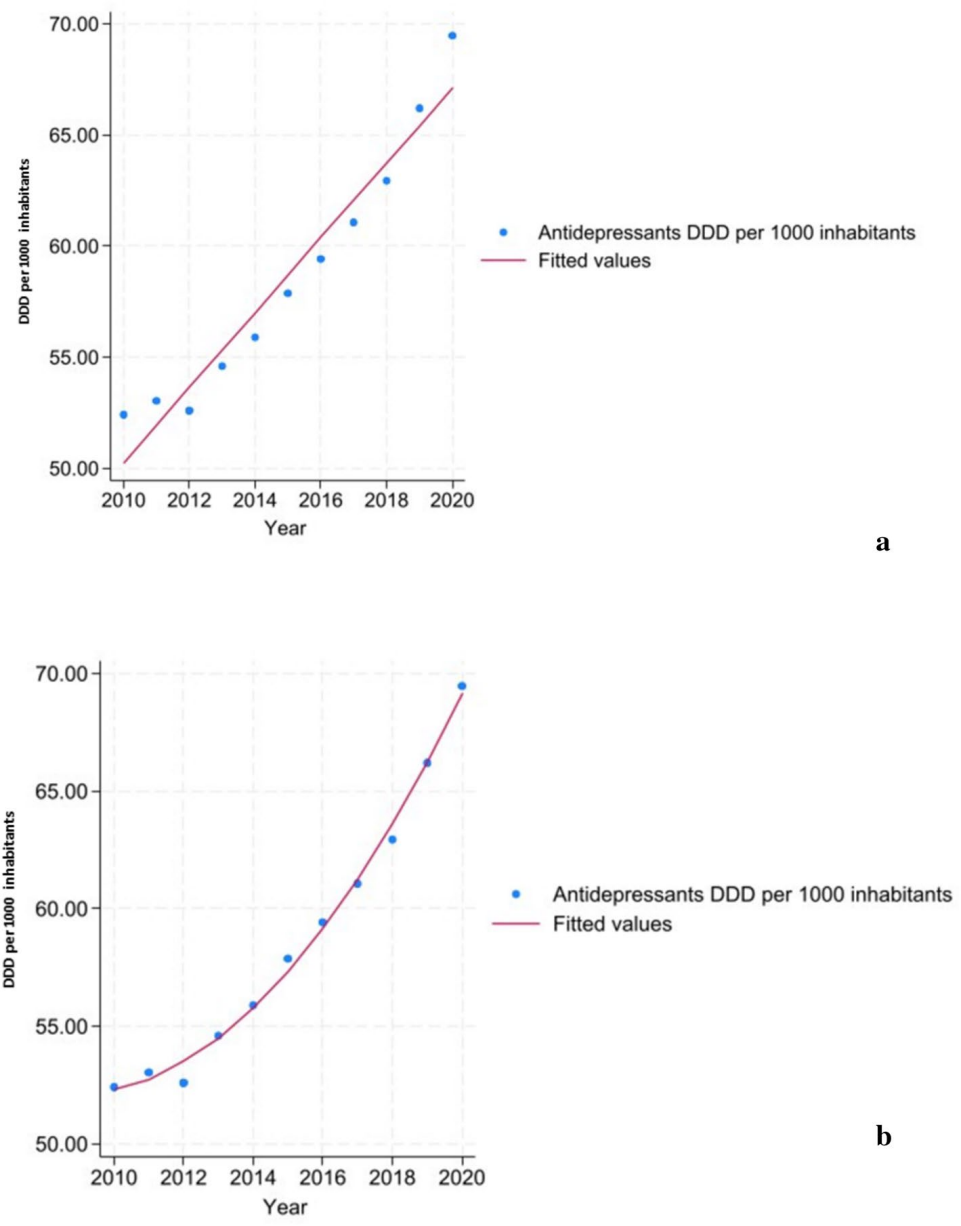
Mean OECD linear (**a**) and quadratic (**b**) trend of antidepressant consumption (DDD per 1000 inhabitants) between 2010–2020. DDD stands for Defined Daily Doses, which is the assumed average maintenance dose per day according to pharmacological indication for adults. The trend (red lines) refers to yearly mean values computed among 30 OECD countries (blue dots). Abbreviations: *DDD* Defined daily dose; *OECD* Organization for Economic Cooperation and Development

**Fig. 3:**
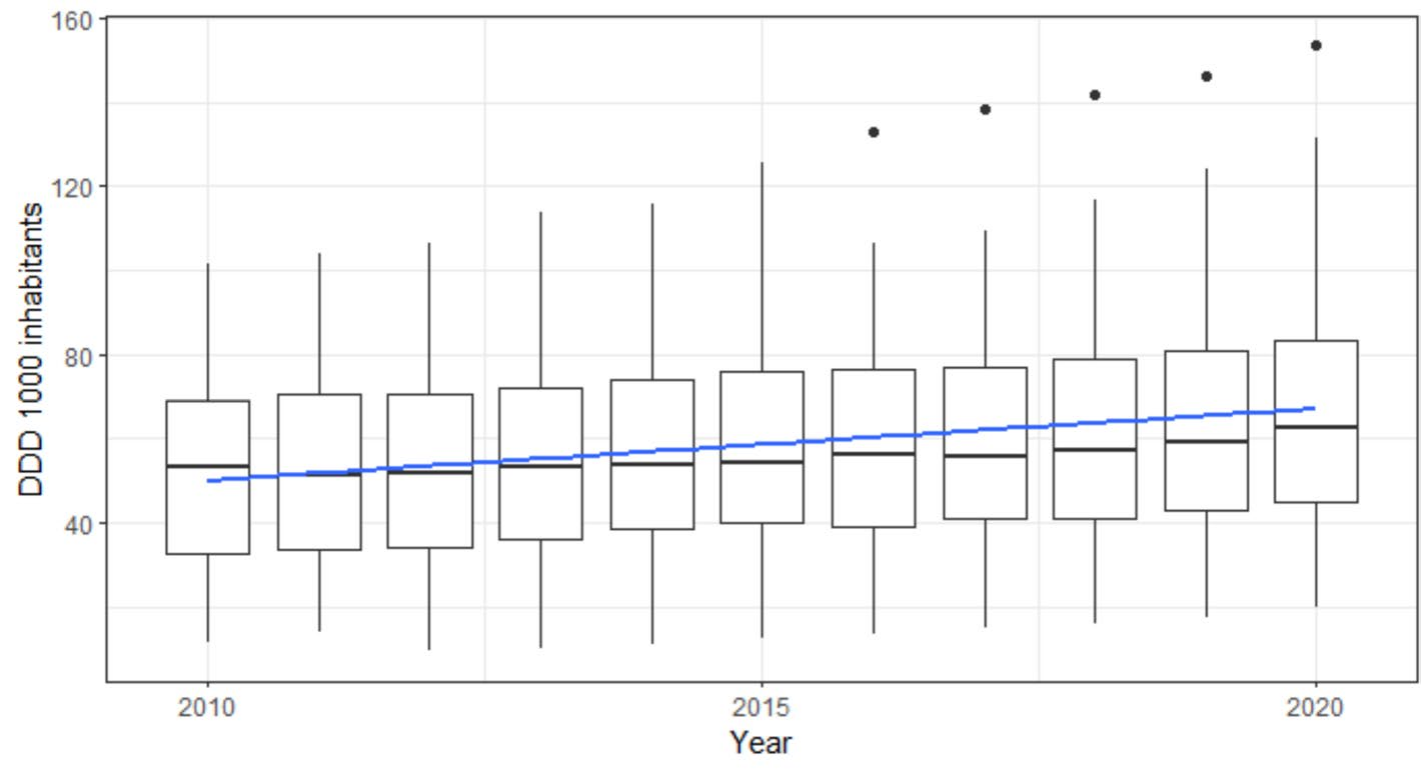
Antidepressants consumption (DDD per 1000 inhabitants) among OECD countries between 2010 and 2020. Box plots represent the annual distribution of DDD among the considered 30 OECD countries. The blue line represents the linear trend of consumption over time. Abbreviations: *DDD* Defined daily dose; *OECD* Organization for Economic Cooperation and Development

The OECD average annual increase in antidepressant consumption was 1.68 DDD per 1,000 inhabitants (linear term coefficient b = 1.68; CI 95% = 1.38, 1.99). However, this increase accelerated over time, as indicated by a significant quadratic term (quadratic coefficient “b-squared” = 0.14; CI 95% = 0.10, 0.17) (Figs.2 and 3).

Over the 11-year period, antidepressant consumption increased in all OECD countries except Denmark and Norway.

### Country-Specific Trends

Three distinct trend patterns were observed based on the positivity of the linear term coefficient (b) and the significance and sign of the quadratic term coefficient, as follows:

1. Linear increasing trend—Countries with a significant positive linear trend (b > 0) and no significant quadratic term, indicating a steady increase in consumption over time.
2. Concave Trend (Slowing Growth)—Countries where the linear term is positive but the quadratic term is significantly negative, indicating a concave trend where the growth rate slows down over time.
3. Convex Trend (Exponential Growth)—Countries where both the linear and quadratic terms are significantly positive, indicating a convex trend where consumption increases at an accelerating pace.

Most countries [14] followed a linear increasing trend (Pattern 1), with b-coefficients ranging from 0.36 (Hungary and Luxembourg) to 3.72 (Australia). Five countries (Austria, Czech Republic, Netherlands, Slovenia, and the United Kingdom) exhibited a concave trend (Pattern 2), suggesting that their antidepressant consumption was approaching a plateau. Seven countries (Canada, Estonia, Finland, Greece, Italy, Latvia, and Portugal) demonstrated an exponential increase (Pattern 3), indicating a convex trend. Denmark, France and Norway deviated from these patterns: Denmark showed a downward trend over time (negative linear coefficient), France had no significant linear trend, and Norway had significant neither linear nor quadratic trend, indicating stable consumption levels. For further details, refer to Table 1 and Supplementary Figures.

**Table 1:** Patterns of consumption: country-specific coefficient linear trend (b-coefficient) and quadratic trend (b-squared coefficient). See the main text for the pattern description

Country	Linear trend			Quadratic trend		
	**b coefficient**	***p*-value**	**CI 95%**	**b-squared**	***p*-value**	**CI 95%**
**Pattern 1**						
Australia	3.72	**0.00**	3.34, 4.10	0.09	0.15	−0.22, 0.04
Belgium	1.53	**0.00**	1.34, 1.73	−0.02	0.56	−0.09, 0.05
Chile	2.75	**0.00**	1.12, 4.37	0.08	0.76	−0.54, 0.71
Costa Rica	1.41	**0.00**	1.05, 1.77	0.04	0.50	−0.09, 0.17
Germany	1.34	**0.00**	1.13, 1.55	−0.05	0.14	−0.12, 0.02
Hungary	0.36	**0.00**	0.30, 0.42	0.01	0.17	−0.01, 0.03
Iceland	5.49	**0.00**	4.99, 5.98	0.07	0.40	−0.11, 0.25
Israel	2.37	**0.00**	2.10, 2.63	0.02	0.59	−0.07, 0.12
Korea	1.26	**0.00**	0.90, 1.63	0.04	0.54	−0.10, 0.18
Lithuania	1.71	**0.00**	1.55, 1.87	0.01	0.75	−0.05, 0.07
Luxembourg	0.36	**0.02**	0.08, 0.65	−0.06	0.19	−0.16, 0.04
Slovak Republic	1.87	**0.00**	1.46, 2.27	0.02	0.79	−0.14, 0.17
Spain	2.59	**0.00**	2.32, 2.86	0.06	0.14	−0.02, 0.15
Sweden	2.97	**0.00**	2.78, 3.15	0.03	0.31	−0.10, 0.04
Turkey	1.34	**0.00**	1.06, 1.62	0.04	0.43	−0.07, 0.14
**Pattern 2**						
Austria	0.71	**0.00**	0.51, 0.91	−0.06	**0.03**	−0.12, −0.01
Czech Republic	2.49	**0.00**	2.35, 2.62	−0.04	**0.03**	−0.08, −0.01
Netherlands	0.74	**0.00**	0.62, 0.86	−0.05	**0.002**	−0.07, −0.02
Slovenia	1.80	**0.00**	1.53, 2.08	−0.10	**0.012**	−0.17, −0.03
United Kingdom	4.74	**0.00**	3.86, 5.61	−0.32	**0.007**	−0.53, −0.11
**Pattern 3**						
Canada	4. 25	**0.00**	3.48, 5.01	0.32	**8,00e-04**	0.18, 0.46
Estonia	2.06	**0.00**	1.91, 2.22	0.06	**0.002**	0.03, 0.09
Finland	1.00	**0.01**	0.31, 1.70	0.30	**4,00e-04**	0.18, 0.41
Greece	2.17	**0.00**	1.54, 2.79	0.28	**0.00**	0.21, 0.36
Italy	0.50	**0.00**	0.36, 0.63	0.06	**1,00e-04**	0.04, 0.08
Latvia	1.05	**0.00**	0.84, 1.26	0.10	**0.00**	0.08, 0.11
Portugal	5.07	**0.00**	4.10, 6.03	0.40	**0.00**	1.46, 2.28
**Other**						
Denmark	−0.66	**0.03**	−1.26, −0.06	0.24	**0.001**	0.13, 0.36
Norway	0.08	0.23	−0.06, 0.23	0.01	0.63	−0.04, 0.07
France	0.12	0.29	−0.13, 0.37	0.10	**0.003**	0.05, 0.15
Mean	1.68	**0.00**	1.38, 1.99	0.14	**0.00**	0.10, 0.17

*CI* Confidence interval, ***p-value*** < *.*05

### Mixed-Effects Model Analysis

Given the hierarchical structure of the data, a multilevel linear regression model was constructed, incorporating a fixed effects for years (2010–2020), and random intercepts and slopes for each country.

The model highlighted a significant fixed effect of years (b = 1.99; 95% CI = 1.39, 2.59), confirming an overall increase in antidepressant consumption across OECD countries. In addition, a substantial impact of clustering was observed, primarily due to variations in baseline antidepressant consumption across countries (Standard Deviation (SD) = 23.1), rather than differences in the rate of increase within each country (SD = 1.65). The conditional R-squared value of 0.99 indicates that 99% of the variance is explained by the combination of fixed and random effects.

In contrast, the marginal R-squared value of 0.05 suggests that only 5% of the variance is explained by fixed effects alone.

## Discussion

This study analyzed antidepressant consumption trends across 30 OECD countries from 2010 to 2020, comparing country-specific rates with the OECD average. Similarly to other studies [17, 18], our findings confirm a general increase in antidepressant use in all countries, except Denmark, France and Norway. Most nations maintained a consistent position relative to the OECD average. Additionally, the multilevel model showed rising antidepressant consumption, though with significant variation across countries. This variability is also observed in the intercept, which in our model represents antidepressant consumption in the first year examined (2010). Inferential statistical analysis showed the annual change in antidepressant consumption, providing information on the scale of the increase. Although antidepressant consumption has mostly grown, there are differences among the country-specific slopes: some are linear, certain are convex, and others are concave.

Countries with high antidepressant use and steep increases included Canada, Iceland, Portugal, and the United Kingdom. In particular, Canada and Portugal exhibited rapid growth, probably due to diverse and joint factors. Improvements in accessibility to mental health services, together with a reduction of stigma about mental disorders, likely represent essential driving forces for both countries [19]. However, some country-specific factors have intensified the phenomenon. In Portugal, mandatory use of the electronic prescription platform inevitably enhanced the surveillance of the administration of psychiatric drugs, thus monitoring the increasing use of antidepressants. In addition, higher recognition of psychiatric disorders, as well as better diagnosis and appropriate treatments through updated guidelines, can have contributed substantially to modifying the upward consumption [19].

Previous studies in Canada have brought to the attention the phenomenon concerning children and adolescents. In 2004, the Federal Drug Administration (FDA) released a black-box label warning as a regulatory action on the prescription and consumption of certain antidepressant drugs for children and adolescents, according to the emerging risk of potentially severe side effects. Nevertheless, a growing trend in North America was observed after a brief slowdown in 2005 and 2009 and during 2012 and 2016. These studies highlight that data refer to physicians’ recommendations, which differ from the prescription and effective administration of antidepressants [20–22].

A 25% rise in the period 2015–2019 and a sharp increase in early 2020, after the official World Health Organization (WHO) declaration of the COVID-19 pandemic, confirmed previous findings from the United Kingdom [23]. Expanded antidepressant indications (e.g., neuropathic pain, urinary incontinence) and increased availability of generic alternatives contributed to higher usage [24–26]. Previous research explained the rising consumption through long-term treated patients, who are often higher consumers of antidepressants [27, 28] compared to those who have short courses accompanied by very high discontinuation rates. More problematic is to comment on the concave trajectories. However, this slope may result from better treatment of patients, perhaps influenced by improved adherence to evidence-based guidelines or longer antidepressant use offset by reduced relapse rates. Also, changing practice approaches by general practitioners (GPs), qualified by empowering their patients, may have modified antidepressant consumption [26]. On the other hand, the concave trend can be viewed as an effect of high demand and consequent lengthened waiting times and crowded waiting lists [27].

Iceland had the highest antidepressant consumption rate in 2010 and 2020 and the steepest increase. Besides the many reasons joined to other countries, this trajectory is probably due to high depression rates for both women and men [28]. High consumption is also likely due to limited psychotherapy access and patient’s perceived effectiveness [29]. Also, regulatory changes in dispensing and reimbursement for SSRIs in 2009 with consequent co-payment reduction allowed larger prescription supplies [30]. Despite increased prescriptions, psychiatric outpatient and inpatient rates continue to rise, raising concerns about public health impacts [31]. In addition, a recent analysis highlighted low mental health status among youth and those who already take psychiatric medicines in Iceland, where, besides medical treatment, only cognitive behavioral psychotherapy is partially subsided by the government. Since the efficacy of psychotherapy is substantially subjective, it is possible that increasing antidepressant consumption is the standard and available way to manage some mental health disorders [32]. Table S1 summarises the main plausible reasons for increasing antidepressant consumption among the OECD countries affected by higher increasing trends.

Rising antidepressant consumption highlights concerns about overmedicalization. While reframing emotional struggles as medical conditions can improve access to care, it also risks overdiagnosis and excessive medication use. To distinguish necessary medicalization from harmful over-medicalization, evaluating whether medicine provides the most safe and appropriate intervention is essential. In the case of psychiatric drugs, this means questioning whether pharmacological treatment is the best solution or whether alternative approaches (e.g., psychotherapy, lifestyle changes, social interventions) could be equally or more effective [33]. In this context, psychiatrisation represents a double-edged phenomenon, improving mental health care but also risks pathologizing normal experiences and fostering dependency on psychiatric interventions. A balanced approach should consider social, political, and cultural frameworks rather than relying solely on medical solutions to address mental distress [34].

While global antidepressant consumption has risen, Denmark is an exception, showing a declining trend over the past 11 years, despite remaining one of the highest-consuming countries. However, Denmark is one of the countries with the highest use of antidepressants, likewise previous findings [14, 35]. Thus, this trend is challenging to interpret, but several factors may contribute. Data reporting issues may lead to potential underestimation, since private healthcare data in Denmark are not systematically included in national registries, unlikely other Scandinavian countries such as Sweden and Norway [36, 37]. Denmark's decentralized healthcare system requires patients to consult a GP first, who may offer conversational therapy rather than medications. Referrals to specialists can take 30 days for diagnosis and up to two months for treatment, potentially delaying antidepressant prescriptions [38]. Another factor could be the "spillover effect" from previous regulatory warnings on antidepressants, which led to a reduction in diagnoses and treatments for depression, particularly among young people and adults [10, 29–32]. This highlights the need for continuous professional education on antidepressant safety and effectiveness. Recognizing this, the Danish Medicines Agency has implemented regulations on how pharmaceutical companies communicate information about medications to healthcare professionals [39]. The decline in antidepressant use may also be linked to broader societal changes. In recent years, Denmark has made significant efforts to reduce stigma around mental health through public awareness campaigns and social media initiatives. Increased openness and discussions about mental health may have influenced treatment-seeking behaviors, potentially contributing to the downward trend in antidepressant prescriptions [38, 39]. The intensive media coverage of mental health issues and alternative treatment approaches may have played a role in shifting public perception and reducing reliance on medication [40].

Norway exhibited no significant change in antidepressant consumption between 2010 and 2020, as neither the linear nor quadratic regression models showed statistical significance. This stability may be attributed to the role of primary healthcare as a regulatory filter for antidepressant prescriptions. In Norway, GPs serve as the first point of contact, prescribing antidepressants when necessary and referring patients to secondary healthcare facilities, including hospital outpatient services or private specialists, for further evaluation. This gatekeeping function likely helps regulate antidepressant use [41]. Despite a rising incidence of depressive disorders among adolescents, particularly girls, over the past decade, treatment rates for new cases of depression remain lower than in other European countries [42]. Antidepressant prescriptions for new diagnoses have remained stable, with many cases not receiving pharmacological treatment [43]. This suggests that Norwegian GPs prioritize non-medical interventions, frequently referring adolescents to mental healthcare services or alternative treatments, such as psychotherapy, which may contribute to containing antidepressant consumption [44].

### Strengths and Limitations

To our knowledge, this is the first study to compare data and trajectories about antidepressant consumption among so many OECD countries based on updated official data. It focuses on the escalation and slowdown of the phenomenon based on country-specific data. The overall assessment of consumption and comparison of consumption rates among countries can facilitate international collaboration in addressing mental health challenges, developing strategies to improve outcomes through investigations on variations in prescribing practices, cultural attitudes towards mental health, the relative efficiency of assistance, and disparities in access to mental health services. Sharing data and evaluating experiences can improve the effectiveness of interventions and prompt public awareness about mental health needs.

Indeed, this study has some limitations. First of all, drug utilisation data presented in DDD give a rough estimate of consumption and not an exact picture of actual use. Indeed, the unit of measurement is DDD, defined as the assumed average maintenance dose per day for a drug used on its main indication in adults. Therapeutic doses for individual patients and patient groups can differ from the DDD as they are based on individual characteristics such as age, weight, ethnic differences, type and severity of disease, and pharmacokinetic considerations.

Other limitations are those of the databases used and are expected in all administrative database studies. Firstly, there are problems related to the data quality, especially the possible lack of accuracy and different data coverage.

In addition, the reported data provide an analysis at the national level, and the state-wide distribution was not analysed.

Finally, while this study provides valuable insights into overall antidepressant consumption trends, further research is needed to analyze the specific classes of antidepressants and their prescribed medical conditions. Different classes vary in their indications, efficacy, and safety profiles. Understanding the distribution of prescriptions among these classes and their association with specific psychiatric and non-psychiatric conditions is essential for optimizing treatment strategies. Future research should also explore prescription patterns, adherence to clinical guidelines, and potential off-label use, ensuring a more comprehensive evaluation of antidepressant utilization in clinical practice.

## Conclusion

This research highlights the increasing trajectories in antidepressant consumption in OECD countries over the past decade, with multiple and different slopes among countries. These trajectories can have potential implications for public health. Indeed, these medications are effective in treating depressive disorders and can improve a patient’s quality of life. In that case, nevertheless, overprescribing, potential adverse reactions, and long-term effects on mental health outcomes are crucial debating issues. More targeted interventions to control the consumption of antidepressants and adequate mental healthcare strategies targeted at the integration of pharmacological and non-pharmacological must be subject to health policy.

## Supplementary Information

Below is the link to the electronic supplementary material.

Supplementary file1 (DOCX 15 KB)

## Data Availability

The current analysis concerns secondary data, already published and available on the OECD health data website (https://data-explorer.oecd.org/).
