# Supplementary material for: International Trends in Antidepressant Consumption: a 10-year Comparative Analysis (2010–2020)
Source: Psychiatr Q. 2025 Mar 3;96(2):241–55. doi: 10.1007/s11126-025-10122-0 (PMC12213856; doi:10.1007/s11126-025-10122-0)
Supplement: Supplementary file 1 — Supplementary file1 (DOCX 15 KB) [file 11126_2025_10122_MOESM1_ESM.docx]

| **TABLE S1.** **Plausible factors for the increase in antidepressant consumption** | **Countries** |
| --- | --- |
| Improved surveillance of antidepressant prescription | Portugal |
| Epidemiology of mental health disorders | UK, Iceland |
| Improved diagnosis (higher awareness of mental health diseases, better recognition of symptoms) and better adherence to evidence-based guidelines | Portugal, UK |
| Reduced costs (introduction of generics, reimbursement policies) | Iceland, UK |
| Lack of accessibility to and availability of alternative treatments (i.e. psychological support) | Iceland |
| Additional indication for antidepressant prescription (i.e. Neuropathic pain, urinary incontinence) | UK |
